# Primary Adenocarcinoma of the Jejunum: Case Report of Rare Small Bowel Neoplasm

**DOI:** 10.7759/cureus.33032

**Published:** 2022-12-28

**Authors:** Fadhel Alherz, Tahseen M Al Omoush, Nasser H Alenezi, Turki F Albalawi, Osama Alsaif

**Affiliations:** 1 General Surgery, Dr. Sulaiman Al-Habib Medical Group, Khobar, SAU; 2 Histopathology, Dr. Sulaiman Al-Habib Medical Group, Khobar, SAU; 3 College of Medicine, Imam Abdulrahman Bin Faisal University, Dhahran, SAU; 4 College of Medicine, King Fahad University Hospital, Dammam, SAU; 5 General Surgery, King Fahad Specialist Hospital, Dammam, SAU

**Keywords:** small bowel obstruction, small bowel resection, neoplasm, primary adenocarcinoma, rare cancer, jejunum adenocarcinoma, small bowel adenocarcinoma

## Abstract

Small bowel adenocarcinoma (SBA) is rare cancer that accounts for less than 2% of all gastrointestinal tract cancers. Usually, the clinical presentation is unspecific, and a patient might complain of nausea, vomiting, abdominal pain, small bowel obstruction, and small bowel bleeding. Because of these unspecific symptoms, the patient might be diagnosed late, from six to ten months, which affects the prognosis. This study contains a case report of a 38-year female with SBA in the jejunum, with unspecific symptoms. She had a history of recurrent admissions due to acute pancreatitis, acute kidney injury, and hyponatremia caused by dehydration. The patient was diagnosed six months after the first symptoms of nausea and vomiting started. The patient underwent exploratory laparotomy for a jejunal stricture mass, and a lymph node was resected. The course after surgery was smooth, and the patient was discharged home on the fourth-day post-surgery. In conclusion, the symptoms of SBA are unspecific and cannot be diagnosed without complete medical histories and lab examinations, making diagnosing SBA challenging.

## Introduction

Small bowel adenocarcinoma (SBA) is rare cancer that accounts for less than 2% of all gastrointestinal tract cancers. Adenocarcinoma accounts for 30-40% of small bowel cancers. Jejunum adenocarcinoma accounts for 29% of SBA [1-3]. The clinical presentation is unspecific; a patient might complain of nausea, vomiting, abdominal pain, small bowel obstruction, and small bowel bleeding [3]. Due to these unspecific symptoms, a patient can receive a late diagnosis, from six to ten months, which affects the prognosis [3]. This paper presents a case of a 38-year-old female with SBA in the jejunum with unspecific symptoms. She had recurrent admissions due to acute pancreatitis, acute kidney injury, and hyponatremia caused by dehydration. A six-month diagnostic delay occurred since the first symptoms of nausea and vomiting presented. The patient underwent exploratory laparotomy for a jejunal stricture mass, and a lymph node was resected. The course after surgery was smooth, and the patient was discharged home on the fourth-day post-surgery.

## Case presentation

This report describes a 38-year-old female who was medically free and had no family history of chronic diseases. Five months ago, she experienced an episodic attack of abdominal pain, nausea, and vomiting; she was treated conservatively in another local hospital. However, her symptoms were not completely relieved and were later aggravated. Two months ago, she was treated for *Helicobacter pylori*. Since then, she has had recurrent admissions due to acute pancreatitis, acute kidney injury, and hyponatremia caused by dehydration. The patient received treatment in this study’s emergency room at Dr. Sulaiman Al-Habib Medical Group for nausea and vomiting for two days. The vomiting was projectile with food content. Associated with a poor appetite, the patient stated that she had an unintended weight loss of 7-8 kg in the last four months. There were no signs of neurological symptoms, fever, urinary symptoms, constipation or diarrhea, skin rashes, or cardiac or respiratory symptoms. The patient was told that she would be admitted for hyponatremia, but she refused admission. One day later, she returned to the outpatient department, having been transferred from another hospital, for further assessment. She has been treated with clexane 40 mg, a multivitamin, via subcutaneous injection once daily. She was admitted for further investigation.

Laboratory tests showed anemia and hyponatremia. The renal function test, liver function test, and coagulation profile were normal (Table 1) [4,5].

**Table 1:** Laboratory investigations.

Test/date	10/5/2022	10/6/2022	10/7/2022	10/8/2022	10/9/2022	10/11/2022	10/13/2022	Normal value
White blood cells	9.27	7.84	7.81	8.16	9.15	8.88	6.95	4-10 × 10^9/L
Hemoglobin	11	11.3	10.7	9.8	10.5	8.3	7.8	12-150 ng/mL (women)
Mean corpuscular volume	85.4	84.4	86.4	85.9	86.6	85.9	87.2	80-100 fL
Platelets	314	326	318	325	320	313	303	150-400 × 10^9/L
Sodium	130	132	130	132	136	135	138	135-145 mmol/L
C-reactive protein	< 1	-	< 1	< 1	1.7	-	241	<5 mg/L
Amylase	51	-	50	-	-	-	-	30-125 U/L
Lipase	48	-	59	-	-	-	-	10-150 U/L
Carbohydrate antigen 19-9	-	-	18.17	-	-	-	-	<37 U/mL

Imaging investigations

**Figure 1:**
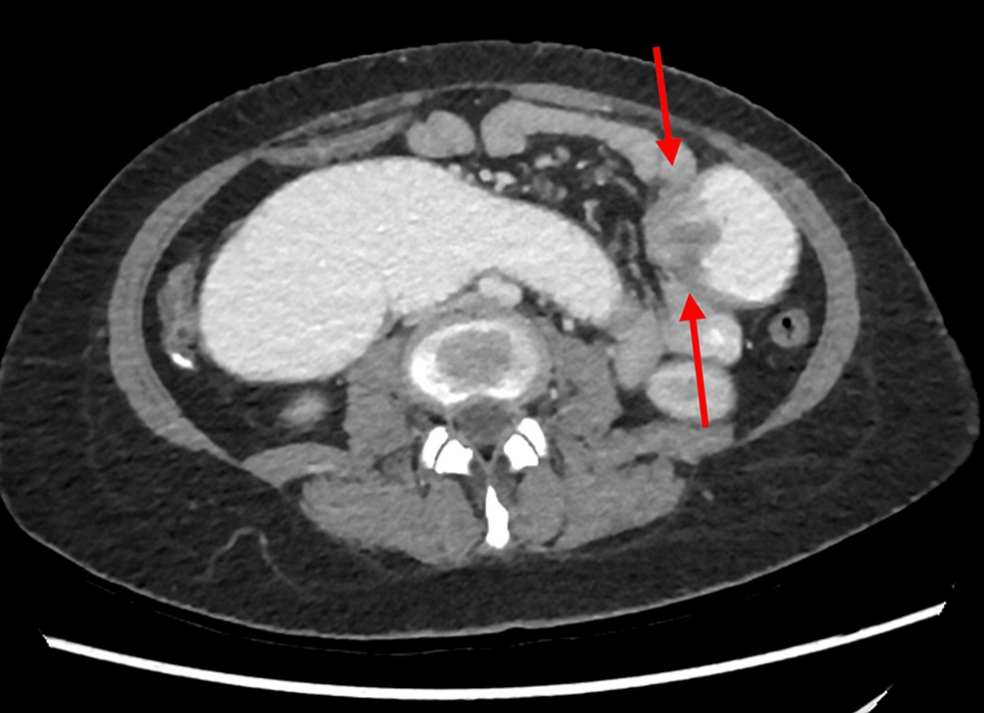
An abdomen computed tomography scan with contrast showing a short segment of irregular circumferential mass involving the proximal part of the jejunum.

**Figure 2:**
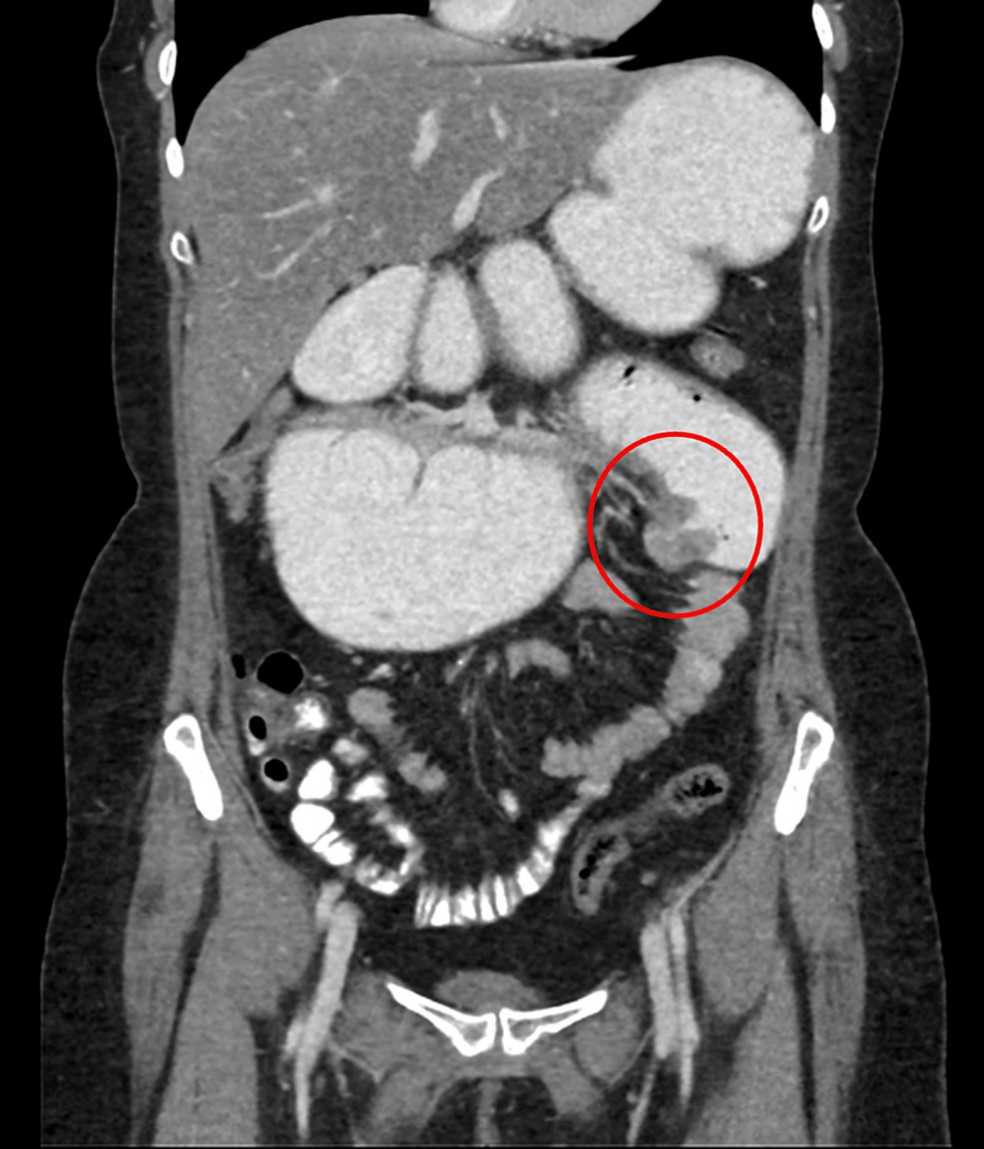
Coronal computed tomography scan showing shouldering of the mass associated with significant dilatation of the proximal jejunum, duodenum, and stomach.

The patient underwent esophagogastroduodenoscopy, which revealed a dilated stomach, duodenum, and proximal jejunum, and a partially obstructing mass was biopsied. A computed tomography (CT) scan of the abdomen with contrast showed a short segment of irregular circumferential mass involving the proximal part of the jejunum (Figure 1). The mass was approximately 2.8 cm in length with a marked narrowed lumen. The proximal edge of the mass was seen to cause shouldering and upward projection and a markedly distended stomach and duodenum (Figure 2). This suggests that the soft tissue mass at the proximal end of the jejunum was likely a neoplastic lesion seen in the esophagogastroduodenoscopy and biopsy. Other than that, the rest of the abdomen CT was normal and unremarkable. The chest CT was also unremarkable, with no evidence of intrathoracic metastasis.

Treatment

The patient underwent exploratory laparotomy for the jejunal stricture mass with a deposit at the mesenteric side of the jejunum resection of the jejunal mass. This was followed by the khorization of the duodenum. The patient received duodenojejunostomy, a side-side anastomosis closure of the duodenotomy with Ethibond stitching, then continuous closure of the mesenteric defect and fixing of the jejunum at the duodenojejunal junction. Her retroperitoneal lymph node was dissected and sent for histopathology.

Histopathology

**Figure 3:**
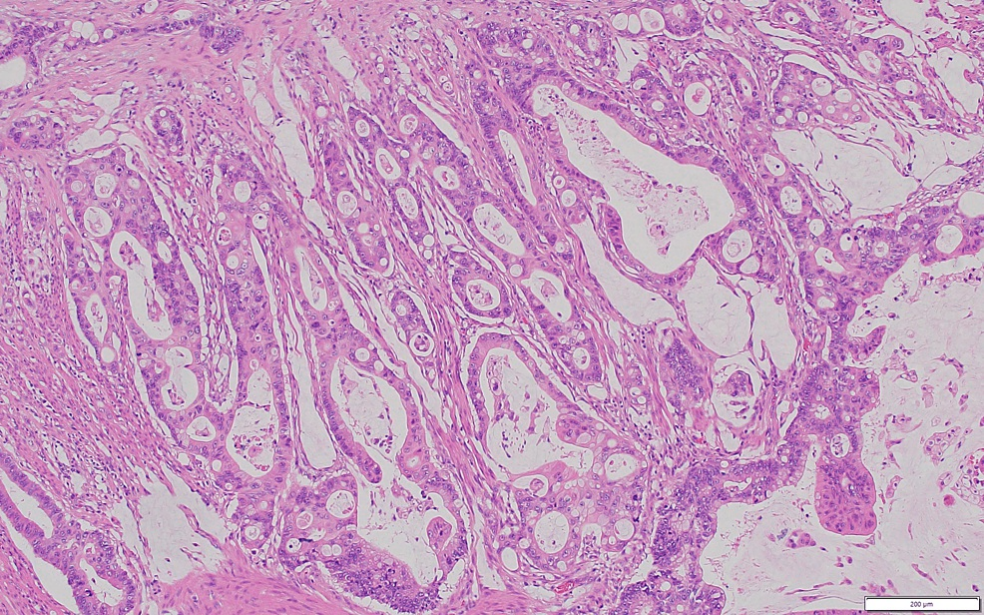
Moderately differentiated adenocarcinoma.

**Figure 4:**
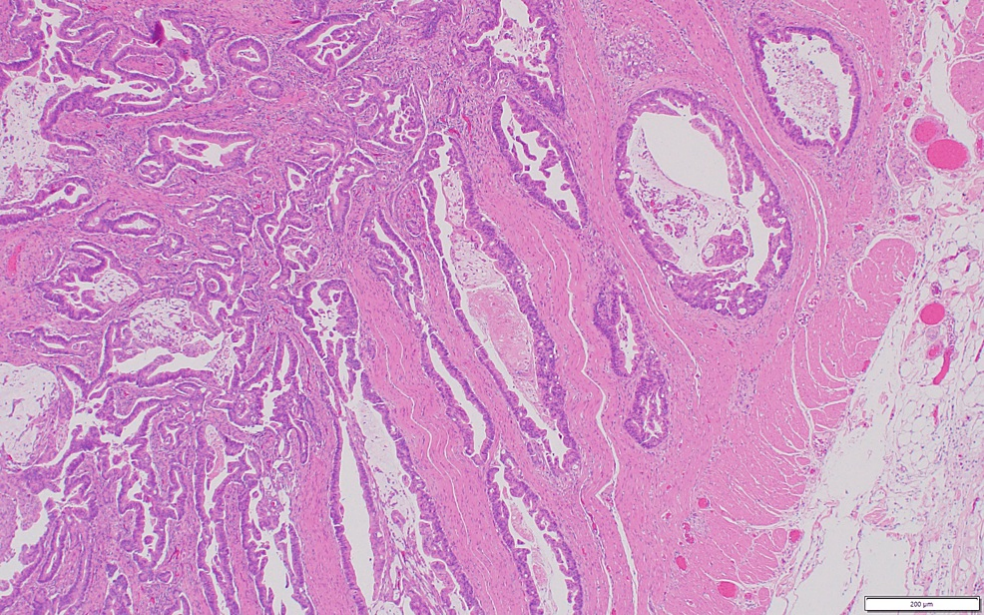
Invading through muscularis propria.

**Figure 5:**
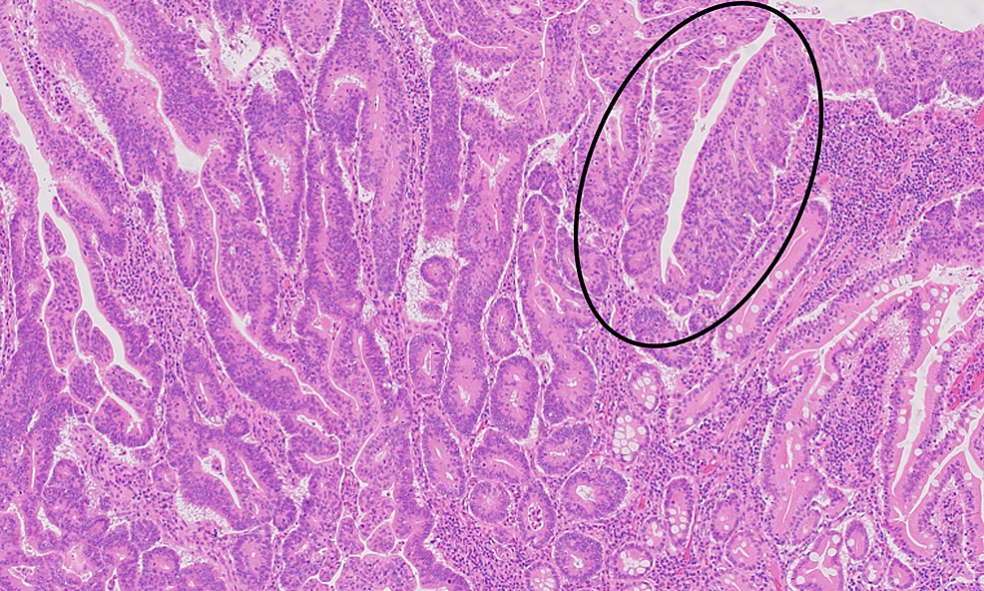
High-grade dysplasia.

**Figure 6:**
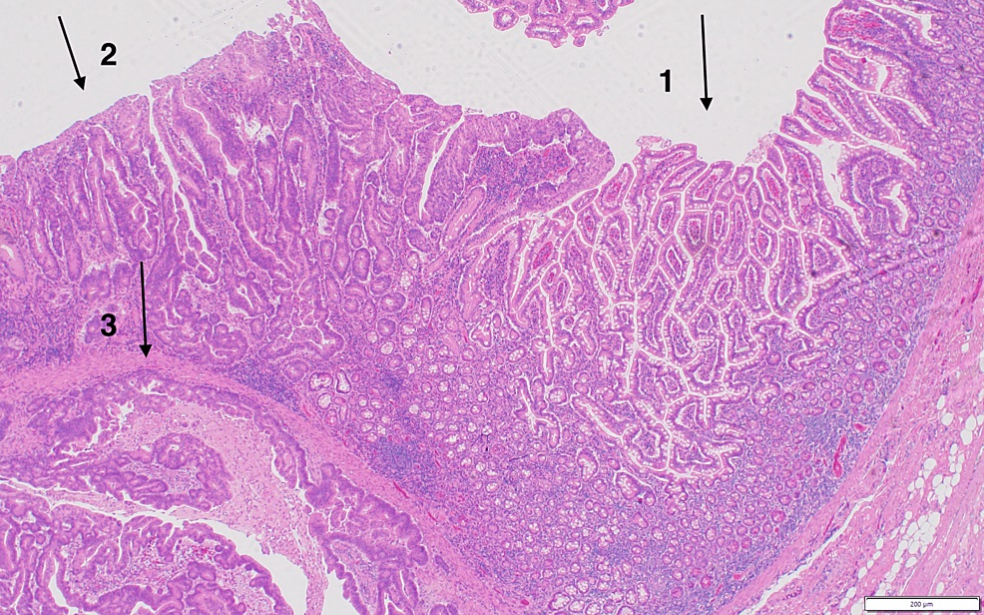
One section of the specimen showed all changes that any small bowel cancer will go through, including (1) mucus of small bowel, (2) high-grade dysplasia, and (3) invasive adenocarcinoma.

Evaluation of the resected specimen revealed a moderately differentiated adenocarcinoma (Figure 3) of the jejunum, 3 cm in size, which invaded through the muscularis propria into the subserosa (Figure 4). All regional lymph nodes were negative for tumor, with 12 nodes examined. All margins were negative for invasive carcinoma, and there was a high-grade dysplasia (Figure 5). The malignancy was staged as T3N0. One section of the specimen showed all the changes that any small bowel cancer undergoes (Figure 6).

Follow-up

The course after surgery was smooth, and the patient was discharged home on the fourth-day post-surgery. The patient was seen in the general surgery clinic 14 days after the surgery. After the histopathology report, it staged as T3N0 no lymph nodes metastasized, CT for chest and abdomen was clear other than this mass. Jejunal and ileal tumors can generally be treated with segmental resection. The surgical margins were clean. Due to the low incidence of SBA, it is still challenging to diagnose and go with a fixed surveillance plan. She was well with no complaints; her eating and bowel movements were normal, the wound was clean, and the clips were removed [6].

## Discussion

SBA is rarer than other gastrointestinal malignancies. Some studies have been conducted regarding its incidence. For example, in Saudi Arabia, a study from 2007 to 2020 included patients diagnosed with SBA, most of them male, and the most common sites were the duodenum (60.5%), jejunum (27.9%), and ilium 7% [7]. Moreover, the American Cancer Society estimates that 10,190 of 310,440 cases in 2017 were small bowel neoplasms [8]. Although 90% of the small bowel comprises absorptive mucosal areas, only 3% of neoplasms arise from the small bowel. Because of small and rare incidences, it affects the clinical attention to SBA. Risk factors for SBA include lifestyle factors such as the excessive consumption of red meat, smoking, and alcohol intake [9,10]. Risk factors also include predisposing diseases, such as Crohn’s and celiac disease, familial adenomatous polyposis, and Peutz-Jeghers syndrome [1]. At this time there weren’t any genetic syndromes evaluated in the patient. No family history of cancers, and the patient was medically free, with no symptoms of Crohn’s or celiac diseases. So there weren’t any syndromes suspected. The symptoms of SBA are not specific, and most cases are chronic presentations with over six months of development, such as in this study’s case report. The symptoms include abdominal discomfort associated with obstruction or lower gastrointestinal bleeding. Moreover, anemia was the most common complaint found in SBA alongside weight loss [11-13]. In this study’s case report, the presented abdominal pains and anemia symptoms were associated with vomiting with weight loss. Regarding the diagnosis, a CT scan can’t be relied upon because it cannot provide an important differential diagnosis for the lesion. However, it helps differentiate benign lesions from malignant ones. In this case, the CT showed an irregular circumferential mass and shouldering of the mass associated with significant dilatation, which meant it could be identified as a malignant lesion. Esophagogastroduodenoscopy was done, and the mass was biopsied. While in treatment, surgical resection with clear margins and a regional lymph node were considered the most suitable choice for SBA [10]. One study showed that most patients with localized SBA underwent curative surgery. In the study, the median overall survival in patients with curative surgery was 94.4 months compared with the no-surgery group, which was 30.1 months. This potentially confirms the pivotal role of surgical resection in managing SBA [13]. Unfortunately, chemotherapy is not frequently used as the primary treatment for small intestinal adenocarcinoma since it does not seem to be extremely responsive to it. Although our patient had stage IIA, T3N0 cancer, which doesn't require this treatment, it may still be administered in some cases, such as in metastasized adenocarcinoma [14].

## Conclusions

The symptoms of SBA are unspecific and cannot be diagnosed until the patient’s history has been obtained alongside complete laboratory examinations, making the diagnosis of SBA challenging. Adenocarcinoma, specifically in the jejunum, is considered rarer than other gastrointestinal malignancies. According to National Comprehensive Cancer Network Guidelines in localized SBA (stages I-III), the primary treatment is surgical resection with clear margins and the removal of regional lymph nodes.
